# Compatibility of a Novel Thrombospondin-1 Analog with Fertility and Pregnancy in a Xenograft Mouse Model of Endometriosis

**DOI:** 10.1371/journal.pone.0121545

**Published:** 2015-03-26

**Authors:** Diane S. Nakamura, Andrew K. Edwards, Soo Hyun Ahn, Richard Thomas, Chandrakant Tayade

**Affiliations:** 1 Department of Biomedical and Molecular Sciences, Queen’s University, Kingston, Canada; 2 Department of Obstetrics and Gynecology, Queen’s University, Kingston, Canada; Center for Cancer Research, National Cancer Institute, UNITED STATES

## Abstract

Endometriosis is a gynecological disease defined by the growth of endometrium outside of the uterus. Although endometriosis contributes to 50% of female infertility cases, medical treatments are incompatible with pregnancy. Angiogenesis, the growth of blood vessels from existing vasculature, plays a crucial role in endometriotic lesion growth and survival. Previously, we demonstrated the effectiveness of thrombospondin-1 analog, ABT-898 (Abbott Laboratories) to inhibit endometriotic lesion vascularization in mice. We have now evaluated the trans-generational implications of ABT-898 treatment before and during mouse pregnancy. We hypothesized that ABT-898 would target lesion vasculature without affecting pregnancy, offspring development, or ovarian and uterine vascularity in mice. Endometriosis was induced using human endometrium in β-estradiol-primed BALB/*c-Rag-2^-/-^Il2rγ^-/-^* mice receiving intraperitoneal injections of ABT-898 (25 mg/kg) or 5% dextrose control for 21 days. Ultrasound assessment of lesion vascularization revealed a reduction in blood flow supplying treated lesions. Excised ABT-898 treated lesions stained for CD31^+^ endothelial cells exhibited a decrease in microvessel density. Following confirmation of estrous cycling, mice were bred and treated with ABT-898 on gestation days 7, 9, 11, 13, 15, 17, and 19. ABT-898 did not affect estrous cycling or pregnancy parameters including litter size across generations and offspring weight gain. Quantification of angiogenic cytokine plasma levels revealed no significant differences between treatment groups. Vimentin staining of the uterus and ovary revealed no observable effects of ABT-898. Similarly, no obvious histological anomalies were observed in the kidney, liver, ovary, or uterus following ABT-898 treatment. These results suggest that ABT-898 effectively inhibit endometriotic lesion vascularization without affecting trans-generational pregnancy outcomes in mice.

## Introduction

Endometriosis is a gynecological disease characterized by the pathological growth of endometrium outside of the uterus [1]. When normal endometrium lining is shed from the uterine wall endometrial fragments can retrogradely migrate through the fallopian tubes and into the pelvic cavity [2–5]. Ectopic implantation of endometrial tissue fragments results in the formation of pathological endometriotic lesions. Common ectopic locations include the ovaries, fallopian tubes, and pelvic peritoneum [1]. Endometriotic lesions can result in the physical blockage of the fallopian tubes and can impair ovarian function contributing to up to 50% of cases of female infertility [1, 6–9]. Approximately 176 million women of reproductive age are affected by endometriosis, many of which are suffering with subfertility or infertility [1, 6–8]. In the United States and Canada alone, $23.8 billion dollars ($22 billion USD, $1.8 billion CAD) are spent annually on the diagnosis and treatment of endometriosis [2–4]. Currently, there are no available treatment options which simultaneously treat endometriosis and infertility [10,11]. Widely-used treatments such as oral contraceptives and gonadotropin-releasing hormone (GnRH) agonists effectively reduce pelvic pain and lesion survival but the anovulatory state induced is incompatible with pregnancy [10,11]. Hence, we sought to demonstrate the efficacy of a novel pharmaceutical agent, ABT-898, to reduce lesion survival while remaining compatible with fertility and pregnancy in mice.

Endometriotic lesion survival is highly dependent on the early establishment of a vascular network to provide nutrients to the pathological tissue [1]. Angiogenesis (the growth of blood vessels from existing vasculature) is a naturally occurring process in eutopic and ectopic tissues [12–14]. Physiological angiogenesis is active during regeneration of the endometrium with newly developed vessels becoming surrounded by a layer of protective pericytes [14]. In comparison, pathological angiogenesis within endometriotic lesions results in the formation of vessels with exposed endothelial cells due to a lack of pericyte recruitment [14,15]. Anti-angiogenic therapies targeting this weakness in pathological vessel integrity may be applicable to the treatment of endometriosis.

Thrombospondin-1 (TSP-1) is a potent regulator of pathological angiogenesis that functions to concurrently inhibit endothelial cell migration and the release of VEGF from the extracellular matrix [16–19]. Anti-angiogenic drugs have been modeled after this peptide and tested against angiogenesis-related diseases such as cancers and endometriosis [20]. Scientists at Abbvie Laboratories have developed a second generation TSP-1-mimetic peptide, ABT-898, that induces the apoptosis of endothelial cells through interactions with CD36 while inhibiting the binding of VEGF to VEGF receptor 2 [21]. Previously, we have demonstrated that ABT-898 inhibits endothelial cell tube formation *in vitro* and lesion vascularization in mice [22]. It is now essential to determine whether ABT-898 specifically targets lesion vascularity without affecting pregnancy. In this study, we examined the implications of ABT-898 treatment before and during pregnancy on trans-generational pregnancy outcomes in a xenograft mouse model of endometriosis.

## Materials and Methods

### Mouse model, ABT-898 treatment regimen, and pregnancy outcomes

Endometriosis was induced in β-estradiol-primed female *Rag-2* ^*-/-*^ *Il2rγ* ^*-/-*^ double-knockout mice on a BALB/c background (*n* = 16) lacking T cells, B cells, and NK cells which were developed and provided by Dr. M. Ito (Central Institute for Experimental Animals, Kawasaki, Japan). Six to eight week-old female mice were given a subcutaneous human β-estradiol implant (15mg/pellet) five days prior to the induction of endometriosis. Sections of non-pathological human endometrium (mean weight, 0.0260 g) collected from the hysterectomized uteri of a patient at Kingston General Hospital, Kingston, ON, Canada, were adhered to the left side of the abdominal wall with 3M Vetbond veterinary adhesive (3M, Milton, ON, Canada) (two sections per mouse). This study was approved by Queen’s University Health Research Ethics Board (Human Ethics approval#ANAT-029-09) and written informed consent was obtained from all the subjects participating in this study. Mice were injected intraperitoneally with 100 μL of 5% dextrose (*n* = 8) (Baxter Corporation) or 100 μL of ABT-898 (Abbott Laboratories, North Chicago, IL, United States) diluted in 5% dextrose (25 mg/kg, *n* = 8) for 21 consecutive days. On the last day of treatment, half of the mice (*n* = 4 per group) were sacrificed using cervical dislocation method approved by the animal care services to excise the lesions. Breeding pairs were created for the remaining mice. Pregnant F0 generation mice were injected with 100 μL of 5% dextrose alone (*n* = 4) or ABT-898 (25 mg/kg, *n* = 4) on gestation days 7, 9, 11, 13, 15, 17, and 19. At reproductive age, F1 generation mice were paired for breeding but received no treatment (*n* = 4 per group). Vaginal cytology samples collected on four consecutive days were staged to identify estrous cycling and reproductive status in F0 and F1 generation mice (*n* = 4 per treatment group). Litter size and offspring weight (at birth, 21 days, and 42 days of age) were recorded. All studies were conducted under barrier husbandry at Queen's University (Kingston, ON, Canada) following protocols approved by the Queen’s University Animal care Committee (Animal Utilization Protocol Number 2013-061-Or-A2). All efforts were made to minimize suffering.

### Assessment of endometriotic lesion vascularization

Three-dimensional images of endometriotic lesions were acquired to assess the volume (mm^3^) of lesions from mice treated with ABT-898 or 5% dextrose. Doppler ultrasound was conducted to assess vascularization within the lesions. Ultrasounds were performed on days 7, 14, and 21 of treatment. Endometriotic lesions excised on day 21 of treatment were snap-frozen in Cryomatrix optimal cutting temperature compound (Thermo Scientific). Cryostat sections (5 μm) of the lesions were fluorescently stained for CD31^+^ endothelial cells with phycoerythrin rat anti-mouse CD31 antibody (33.3 μg/mL) (BD Biosciences). ImageJ Pro Plus software version 6.0 (NIH, Bethesda, MD) was used to quantify CD31^+^ endothelial cell fluorescence.

### Assessment of physiological angiogenesis and organ histology

Plasma was extracted from mouse peripheral blood samples collected on days 7, 14, and 21 of treatment (*n* = 8 per group at each time-point). Nine cytokines (interleukin-15, IL-15; interleukin-18, IL-18; basic fibroblast growth factor, bFGF; leukemia inhibitory factor, LIF; macrophage-colony stimulating factor, M-CSF; monokine induced by gamma interferon, MIG; macrophage inflammatory protein-2, MIP-2; platelet-derived growth factor-BB, PDGF-BB; and vascular endothelial growth factor, VEGF) known to be involved in angiogenesis were quantified in plasma using a Bio-Plex Pro mouse cytokine 9-plex assay and standard protocol (Bio-Rad Laboratories). To evaluate the integrity of endothelial and stromal cells, the uteri and ovaries of mice treated with ABT-898 or 5% dextrose were paraffin-embedded, sectioned (5 μm), and stained with vimentin primary antibody (1:500) (Ab7388; Abcam), cytokeratin primary antibody (1: 500) Z0662; DAKO)) and secondary polyclonal goat anti-rabbit biotinylated antibody (1:1000, (DAKO cytomation). The liver, kidney, ovary and uterus harvested from experimental mice on day 21 of treatment with ABT-898 or 5% dextrose were paraffin-embedded, sectioned (5 μm), and stained with hematoxylin and eosin and periodic acid schiff’s stain using a standard protocol.

### Statistical analysis

Analysis of cytokine data and offspring weight gain was performed using a repeated measures two-way ANOVA with a Tukey post-test. Litter size data were analyzed using a one-way ANOVA with a Tukey post-test. CD31^+^ fluorescence data were analyzed with an unpaired t-test. All data samples passed normal distribution and variance tests. A *P*-value < 0.05 was considered significant. All statistical analyses were performed using SigmaStat 3.0 software (Systat Software, San Jose, CA).

## Results

### ABT-898 affects the vascularization of human endometriotic lesions in a mouse model

**Fig 1:**
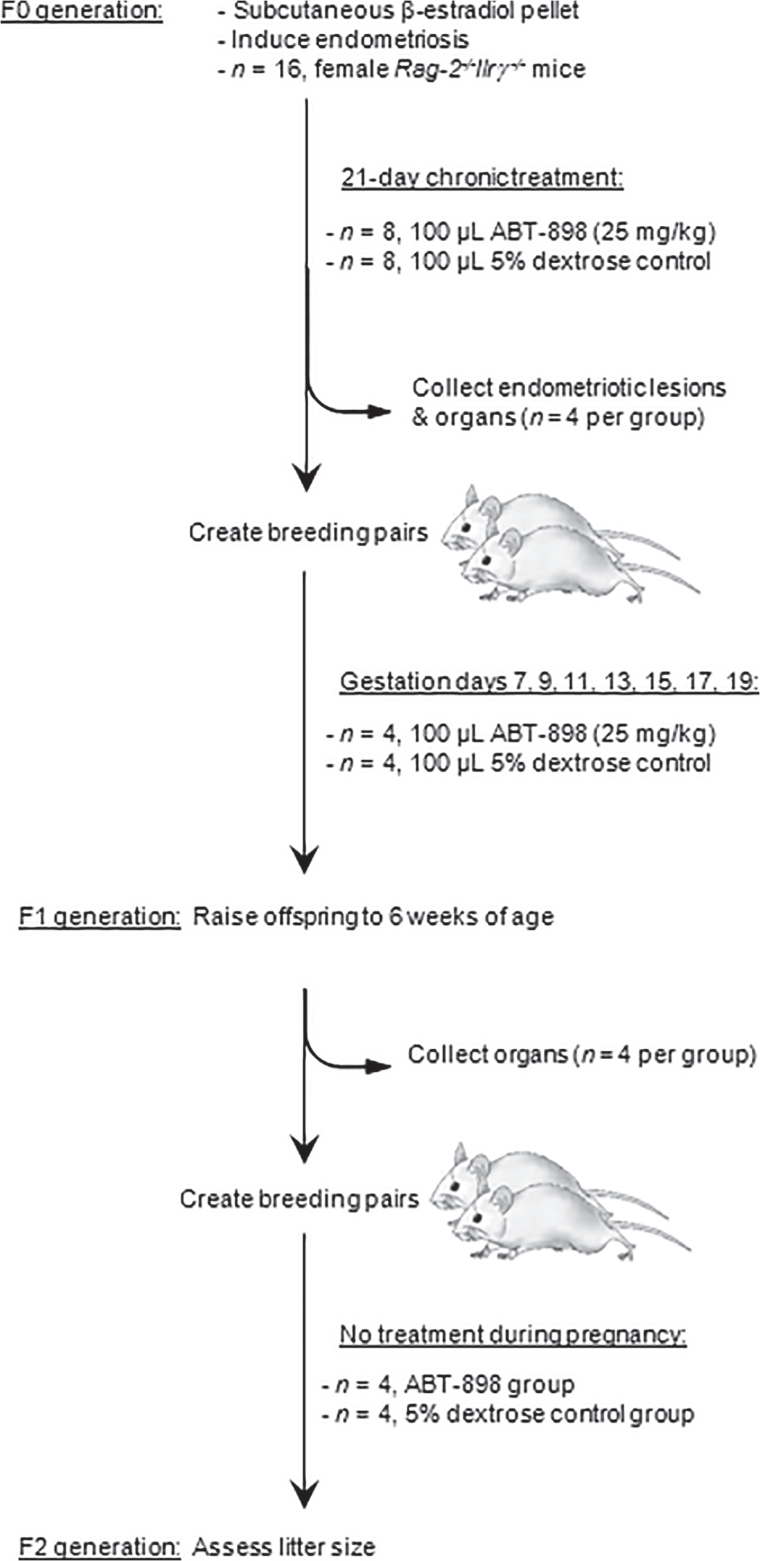
Experimental outline of a trans-generational study.

Female *Rag-2-/- Il2rγ-/-* mice were randomly assigned to a treatment group receiving either anti-angiogenic ABT-898 or 5% dextrose control for 21 consecutive days (Fig. 1).

Twenty-one-day-release human β-estradiol pellets were subcutaneously implanted in female *Rag-2* ^*-/-*^ *Il2rγ* ^*-/-*^ double-knockout mice on a BALB/c background (*n* = 16, F0 generation). The next day, endometriosis was surgically induced by adhering sections of eutopic human endometrium to the abdominal wall. Following the induction of endometriosis, mice were divided into two treatment regimens: mice were intraperitoneally injected with 100 μL of ABT-898 in 5% dextrose (25 mg/kg, *n* = 8) or 100 μL of 5% dextrose alone (*n* = 8) for 21 consecutive days. On the final day of treatment *n* = 4 mice per group were euthanized for the collection of endometriotic lesions and organs. The remaining mice in each group were paired for breeding. Pregnant mice received injections of ABT-898 or 5% dextrose (as previously described) on gestation days 7, 9, 11, 13, 15, 17, and 19. Offspring (F1 generation) were raised to reproductive age (6–7 weeks) and breeding pairs were created to assess reproductive status while receiving no treatment during pregnancy. Offspring of the F1 generation (F2 generation) were also raised to reproductive age. Vaginal cytology was analyzed to identify estrous cycling before creating breeding pairs. All offspring were weighed at birth, 21 days, and 42 days post-partum. Organs were harvested from F0 and F1 generations to histologically assess organ structure and pathology.

**Fig 2:**
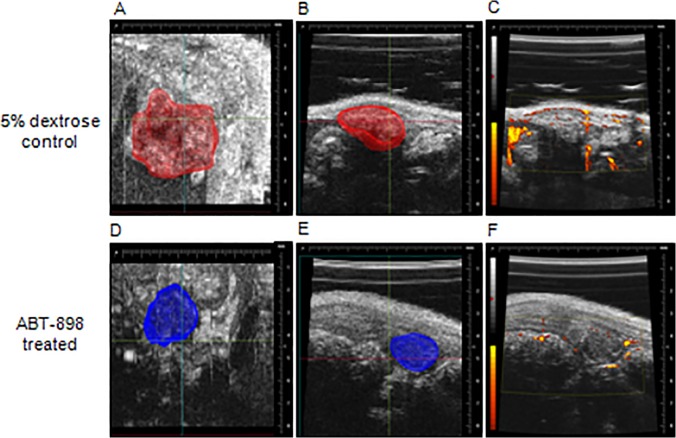
Ultrasound images of human endometriotic lesions.

On days 7, 14, and 21 of treatment ultrasounds were conducted to qualitatively investigate the vascularization and development of endometriotic lesions *in vivo*. Three-dimensional visualizations of the endometriotic lesions acquired by ultrasound revealed no qualitative changes in lesion volume in the ABT-898 treated mice compared to 5% dextrose controls (Fig. 2A, B, D, E). Concurrently, Doppler ultrasound of the lesions revealed a reduction in blood flow supplying the endometriotic lesions of mice treated with ABT-898, thus, highlighting the anti-angiogenic effects of this peptide inhibitor (Fig. 2C, F).

Three-dimensional ultrasound images were compiled to qualitatively assess endometriotic lesion volume *in vivo*. Lesion size was comparable between mice treated with 5% dextrose (A, top profile; B, side profile) and ABT-898 treated mice (D, top profile; E, side profile). (C, F) Doppler ultrasounds revealed a qualitative reduction in blood flow supplying lesions following chronic treatment with ABT-898.

**Fig 3:**
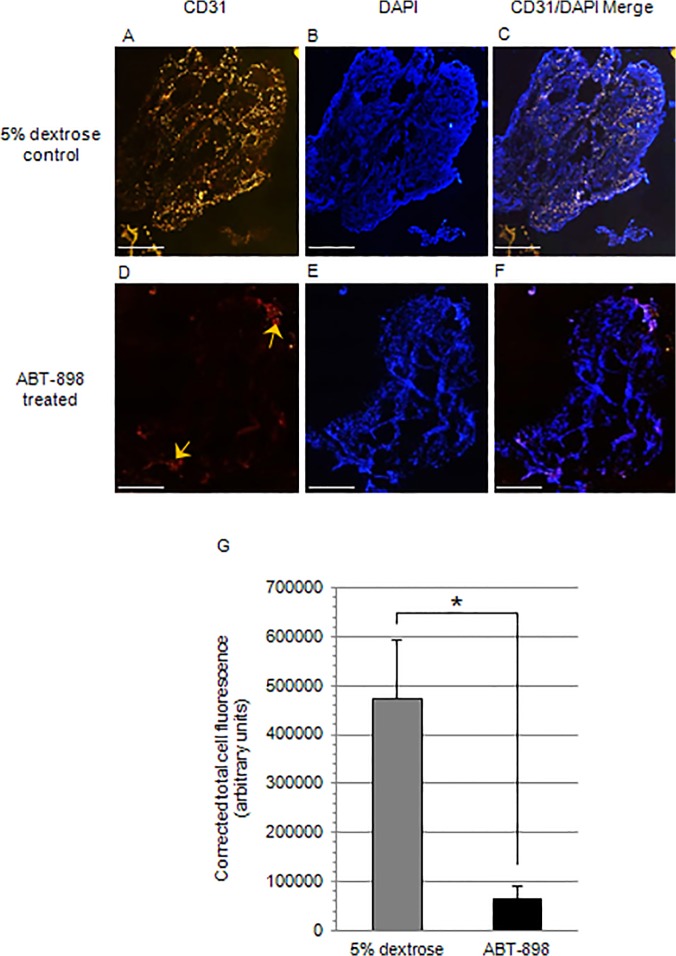
CD31+ endothelial cells within human endometriotic lesions.

On the final day of treatment, mice from ABT-898 and control groups were sacrificed and endometriotic lesions were harvested. Microvessel density within the lesion was visualized with CD31 immunostaining of the endothelial cells lining the blood vessels. The degree of CD31+ staining was significantly greater in the lesions collected from mice treated with 5% dextrose (Fig. 3A-G). These findings support the anti-angiogenic role of ABT-898 in reducing the neovascularization of endometriotic lesions *in vivo*.

Human endometriotic lesions from mice chronically treated with ABT-898 or 5% dextrose were harvested for immunofluorescent staining of CD31+ endothelial cells. (A-C) Lesions harvested from mice chronically treated with 5% dextrose or (D-F) ABT-898 were immunohistochemically stained for CD31^+^ endothelial cells (yellow arrows) with rat phycoerythrin-conjugated anti-mouse CD31 primary antibody and DAPI. (G) Quantification of CD31+ staining revealed a reduction in microvessel density within lesions treated with ABT-898 compared to controls. *P < 0.05. Scale bar = 100 μm. Magnification 400x.

### Trans-generational evaluation of the effects of ABT-898 on mouse pregnancy

Current treatment options for endometriosis target the minimization of chronic pain through methods which preclude pregnancy [1]. We have previously shown that treatment with ABT-898 does not interfere with implantation site structure and vascularization on gestation day 12 [22]. In this study, for the first time, we have assessed the effects of ABT-898 on full-term mouse pregnancies and offspring development in a trans-generational study.

**Fig 4:**
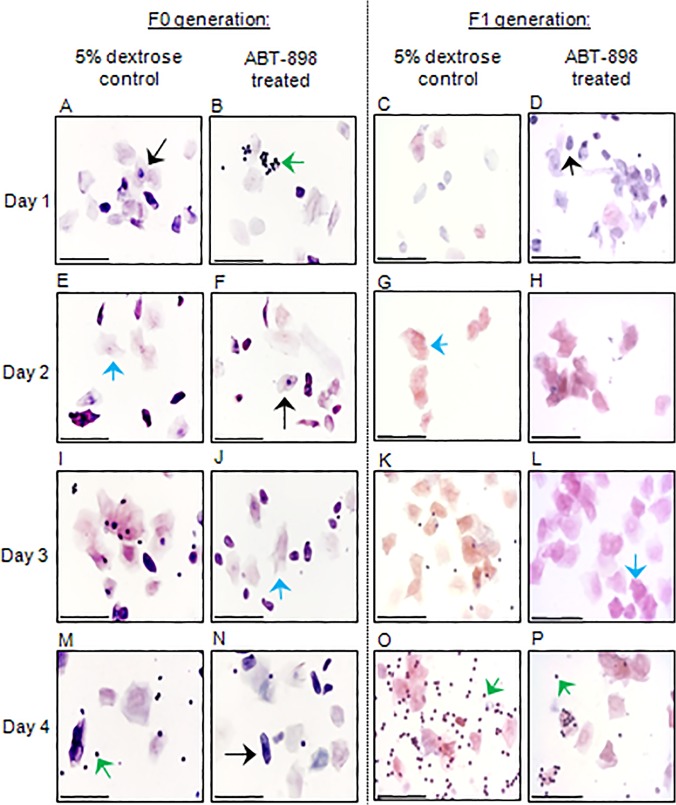
Mouse estrous cycling.

In the first stage of our study, endometriosis was induced in F0 generation mice followed by chronic treatment with ABT-898 or 5% dextrose for 21 days (Fig. 1). Vaginal cell samples collected from F0 and F1 generation mice were staged to identify estrous cycling. The four stages of estrous were identified in all treatment groups within the F0 generation (Fig. 4A, B, E, F, I, J, M, N) and F1 generation (Fig. 4C, D, G, H, K, L, O, P) suggesting that ABT-898 does not affect estrous cycling in chronically treated mice or offspring exposed to ABT-898 *in utero*.

In preparation for breeding, vaginal cell samples collected on four consecutive days from F0 generation mice treated with 5% dextrose (A, E, I, M) or ABT-898 (B, F, J, N) were analyzed by observing the ratio of leukocytes (green arrows), cornified cells (blue arrows), and epithelial cells (black arrows) using a hematoxylin and eosin stain. The vaginal aspirate of the F1 generation from mothers treated with 5% dextrose (C, G, K, O) or ABT-898 (D, H, L, P) were also analyzed prior to breeding. Both F0 and F1 generations progressed normally through diestrus, proestrus, estrus, and metestrus. Scale bar = 75 μm, Magnification 400x.

**Fig 5:**
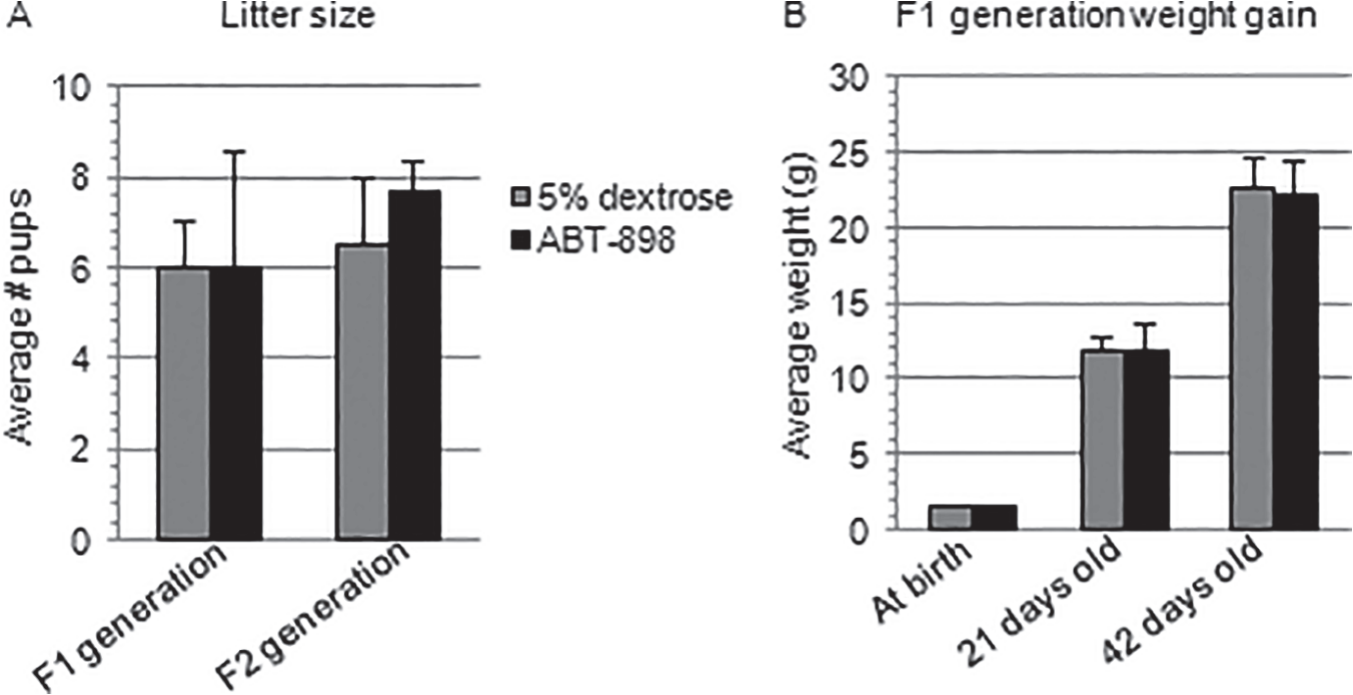
Trans-generational pregnancy outcomes.

F0 generation mice treated with ABT-898 or 5% dextrose for 21 consecutive days were able to achieve a pregnant state irrespective of experimental group. Pregnant F0 generation mice that received injections of ABT-898 or 5% dextrose during gestation yielded litters of similar size (Fig. 5A). Furthermore, F1 generation mice gained weight at a comparable rate irrespective of treatment when weighed at birth, 21 days, and 42 days post-partum (Fig. 5B). To identify trans-generational effects of ABT-898 on pregnancy outcomes, F1 generation pups were bred and received no intervention during pregnancy. Similarly, the number of F2 generation pups per litter was not significantly different between treatment groups (Fig. 5A).

F0 generation female mice received injections of ABT-898 or 5% dextrose for 21 consecutive days before breeding and during pregnancy on gestation days 7, 9, 11, 13, 15, 17, and 19. No significance was found between the litter sizes of the F0 generation experimental groups (A). Breeding pairs were created within the F1 generation and (A) average litter size was observed to be similar in the ABT-898 and 5% dextrose control groups (F2 generation).The F1 generation pups were weighed at birth, 21 days, and 42 days post-partum (B). No significant differences were observed between treatment groups.

### Effects of ABT-898 on physiological angiogenesis and angiogenic cytokine profiles

**Fig 6:**
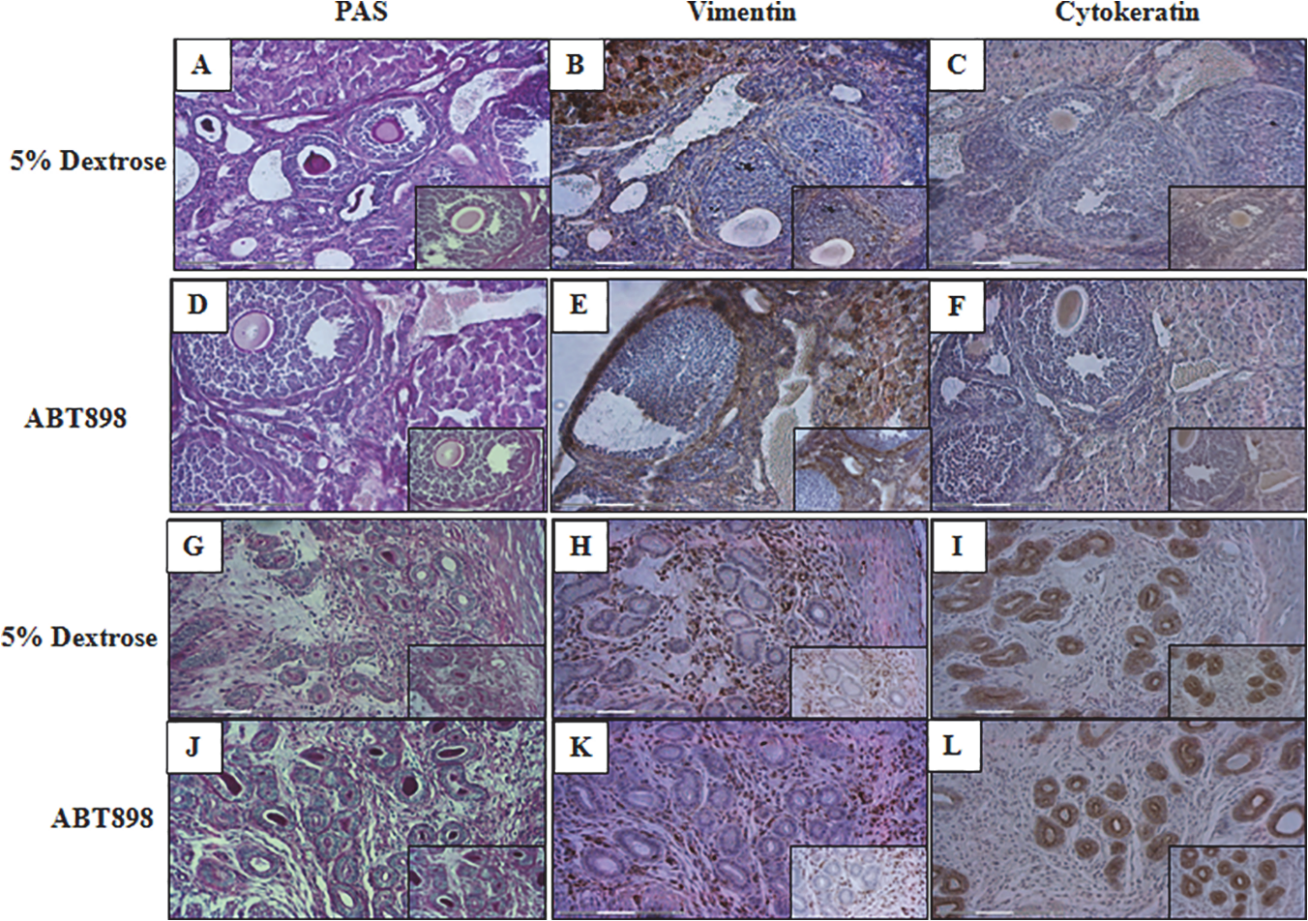
Vimentin staining of the ovaries and uterus.

Angiogenesis is crucial in physiological processes including ovarian follicular development and pregnancy. Consequently, we examined the effects of antiangiogenic ABT-898 on normal physiological angiogenesis in the uterus. Uteri were harvested from F0 generation mice chronically treated with ABT-898 or 5% dextrose. We used serial sections of the ovaries and uteri obtained from 5% dextrose control and ABT-898 treated mice and stained with hematoxylin and eosin and periodic acid schiff’s stain, vimentin immunohistochemistry to assess proliferation and differentiation of stromal and endothelial cells and cytokeratin immunostaining to assess integrity of epithelial cells. Vimentin is an intermediate filament protein expressed by mesenchymal cells types including endothelial and stromal cells. Vimentin staining was found in stromal and endothelial cells but not in the epithelial compartments. Cytokeratin is an intermediate filament found in the intracytoplasmic cytoskeleton of epithelium. The relative level of stromal and endothelial and epithelial cell staining was comparable between the uteri and ovaries of mice treated with 5% dextrose (Fig. 6) and ABT-898. The preservation of cellular and vascular integrity within the uteri and ovaries exposed to ABT-898 provides evidence that physiological angiogenesis may not be impaired by the anti-angiogenic effects of ABT-898.

Serial sections of ovaries and uteri obtained from 5% dextrose control and ABT-898 treated mice were stained with periodic acid schiff’s reagent (A,D,G,J), vimentin immunostaining (B,E,H,K) and cytokerating immunostaining (C,F,I,L). No histological alterations could be detected with periodic acid schiff’s reagent staining in the ovaries and uteri obtained from both ABT-898 treated and control mice. Positive staining in the stromal and endothelial cells is clearly visible in response to vimentin staining in the ovaries (B, E) and uteri (H, K) in 5% dextrose control and ABT-898 treatment groups, respectively. Staining with cytokeratin shows imunoreactivity of epithelial cells in the ovaries (C,F) and uterine glands (I, L) in 5% dextrose control and ABT-898 treatment groups, respectively. Magnification 200x (Images A-L, inset magnification 400x).

**Fig 7:**
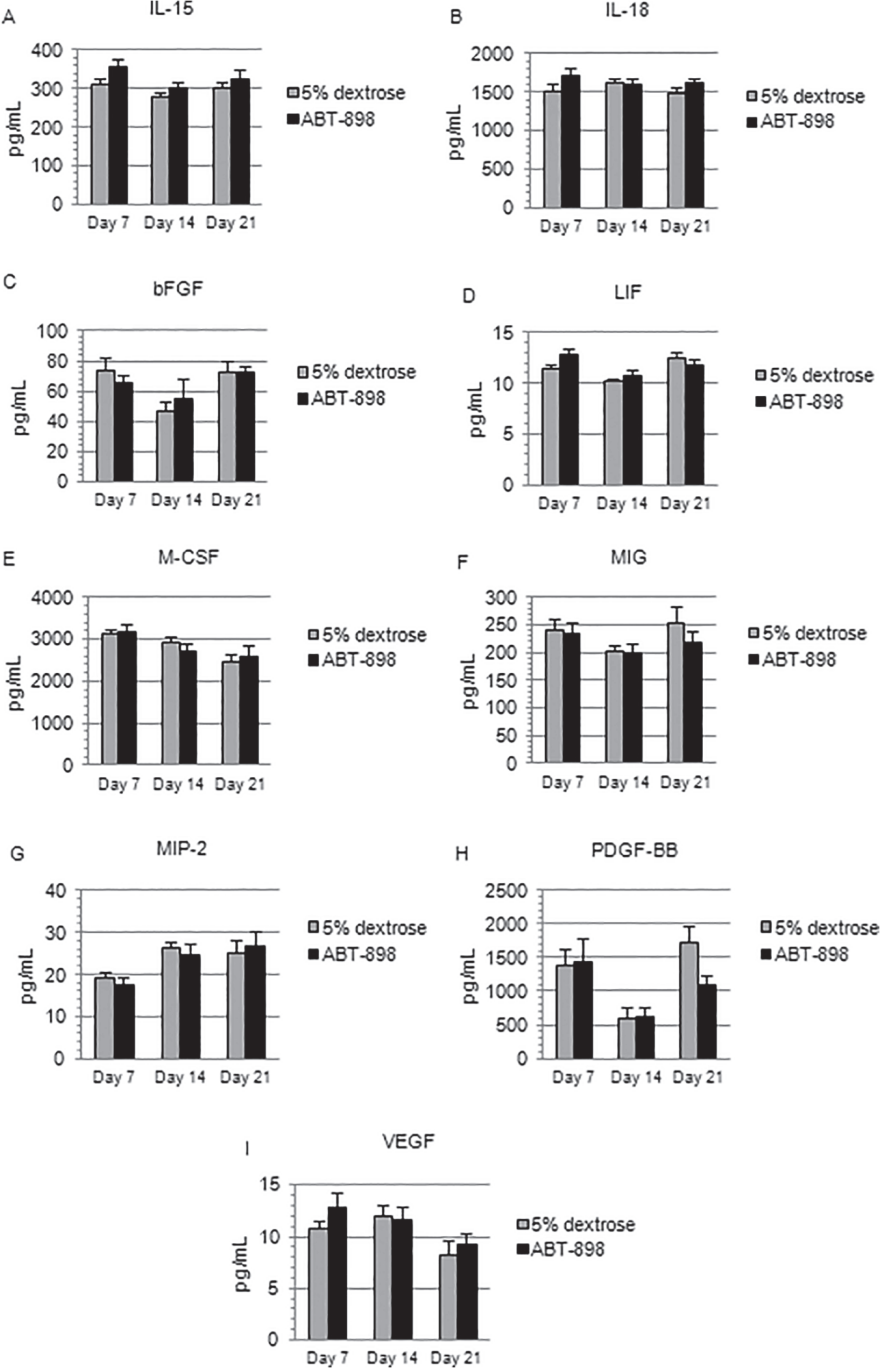
Plasma levels of angiogenic cytokines. (A) IL-15, (B) IL-18, (C) bFGF, (D) LIF, (E) M-CSF, (F) MIG, (G) MIP-2, (H) PDGF-BB, and (I) VEGF plasma levels were analyzed on days 7, 14, and 21 of a 21-day treatment regimen. No significant differences were detected between ABT-898 treated mice and 5% dextrose controls on days 7, 14, or 21. Data are expressed as means ± SD.

To quantitatively assess the effects of ABT-898 on physiological angiogenesis, we analyzed the plasma levels of nine cytokines (IL-15, IL-18, bFGF, LIF, M-CSF, MIG, MIP-2, PDGF-BB, VEGF) involved in angiogenesis during chronic treatment with ABT-898 or 5% dextrose. Plasma was extracted from peripheral blood samples collected on days 7, 14, and 21 of treatment. At each time point, no significant differences in IL-15, IL-18, bFGF, LIF, M-CSF, MIG, MIP-2, PDGF-BB, or VEGF levels were observed between experimental groups (Fig. 7A-I). Therefore, ABT-898 may not affect the plasma levels of cytokines regulating angiogenic processes and thus may not influence physiological angiogenesis.

### Effects of ABT-898 on kidney, liver, ovary and uterus histology

**Fig 8:**
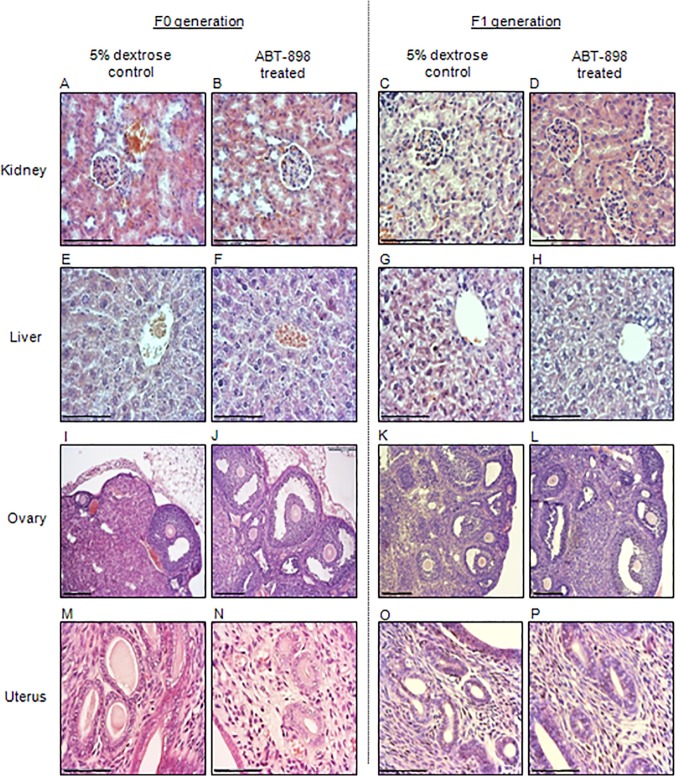
Kidney, liver, ovary, and uterus histology.

Anti-angiogenic drugs such as thalidomide have been associated with a high rate of organ dysgenesis [23]; therefore, we evaluated the effects of ABT-898 on kidney, liver, ovary, and uterus histology. Following a 21-day treatment regimen, F0 generation mice receiving injections of ABT-898 and 5% dextrose were sacrificed for the collection of the liver, kidney, ovaries, and uterus. Mice from the F1 generation were also sacrificed for harvesting of the liver, kidney, ovaries, and uterus. Sections were stained with hematoxylin and eosin and were examined for structural damage symptomatic of organ pathologies. Kidney sections from F0 and F1 generations exhibited similar numbers of glomeruli oriented within the Bowman’s capsule as well as structurally normal renal tubules irrespective of treatment (Fig. 8, A-D). In the liver, the proper organization of hepatic cords separated by sinusoids was observed in mice treated with ABT-898 or 5% dextrose (Fig. 8, E-H). Primary and secondary follicles were found in the ovaries of mice from both of the experimental groups and generations (Fig. 8, I-L) and no architectural damage was observed. Lastly, staining of the collected uteri revealed uterine glands and vessels in both ABT-898 and 5% dextrose treated mice (Fig. 8, M-P).

Hematoxylin and eosin staining of kidney, liver, ovary, and uterus sections from F0 generation mice treated with (A, E, I, M) 5% dextrose or (B, F, J, N) ABT-898 revealed no obvious structural abnormalities. F1 generation mice born to mothers treated with (C, G, K, O) 5% dextrose or (D, H, L, P) ABT-898 during pregnancy revealed normal organ histology. Scale bar = 75 μm. Magnification 400x (kidney, liver, uterus), 100x (ovary).

Overall, examination of tissue sections revealed no obvious structural differences between treatment groups in both F0 and F1 generations providing evidence that ABT-898 may not affect the histological structure of the kidney, liver, ovary, or uterus.

## Discussion

Endometriosis is a primary cause of female infertility contributing to approximately 50% of cases [1,24]. The treatment of endometriosis-related infertility is complex due to the estrogen-dependent nature of the disease [25]. Commonly prescribed therapies modulate the expression of sex steroids to induce an anovulatory state in patients and, therefore, are not recommended for the treatment of endometriosis-associated infertility [10,11,26,27]. In addition, existing therapies provide temporary, short-term relief from pelvic pain and other related symptoms. No studies to date have assessed the effects of anti-angiogenic therapies on pregnancy or fertility across generations. In this novel study, we investigated the effects of TSP-1-mimetic, ABT-898, on reproductive outcomes in a xenograft murine model of endometriosis. Importantly, we have demonstrated that ABT-898 does not impair fertility or pregnancy outcomes across three generations of mice.

The growth and survival of endometriotic lesions may be impaired through the inhibition of pathological angiogenesis to reduce the supply of nutrients to the ectopic tissue [22]. ABT-898 uniquely induces the apoptosis of endothelial cells via the CD36 pathway while sequestering pro-angiogenic VEGF to inhibit endothelial cell proliferation and migration [21,22,28]. In agreement with our previous findings, we observed a significant reduction in the number of CD31+ endothelial cells within treated lesions although Doppler ultrasound revealed no significant changes in lesion volume after treatment [22]. It is possible that we were unable to measure accurate changes in the lesion volume during the treatment period using Doppler ultrasound approach. It was essential for us to confirm that ABT-898 was functioning to reduce lesion vascularization before proceeding to pregnancy trials. Unlike in our previous study, we have treated F0 generation mice during pregnancy to evaluate the effects of ABT-898 *in utero*. Since estrous cycling and reproductive status were maintained in both F0 and F1 generations, we can speculate that ABT-898 does not impair fertility. Previously, we identified no histological defects in gestation day 12 implantation sites and implantation site numbers were unaffected from mothers treated with ABT-898. In extension, we have now shown that ABT-898 exposure before and during pregnancy does not affect litter size or weight gain of offspring in F1 and F2 generations. These data suggest that treatment with ABT-898 effectively reduces lesion vasculature but may not impair trans-generational reproductive status, pregnancy rates, or offspring development.

The importance of identifying teratogenic effects of agents related to pregnancy was highlighted by the deleterious outcomes of using thalidomide to treat morning sickness [23]. Although tested on rodents, malformations were only observed in rabbits and primates due to differences in the activation of liver microsomes [23]. Nonetheless, in this study we utilized a xenograft mouse model. Immunodeficient strains of rabbit are poorly defined and autologous animal models of endometriosis exclude factors of human endometrium itself that may play a role in disease pathogenesis. Immune system plays an important role in endometriosis lesion establishment and disease progression. An Autologous rodent model where endometrial fragments from the same mouse are transplanted peritoneally with an intact immune system provides an opportunity to better understand pathophysiology of endometriosis. This aspect is lost in xenotransplant models involving immunodeficient mice. In the current study we used alymphoid mice as this was the only available model system to investigate human endometrial responses to anti-angiogenic compounds such as ABT-898. Available literature suggest that the human endometrial fragments implanted in immunodeficient mice form endometriotic-like lesions, that has several macroscopic and histological similarities with the lesions found in patients, steroid responsiveness and vascularization supporting the lesions. However, one of the limitations of this mouse model is the inability to investigate direct effects if ABT-898 on immune cell functions, as these mice lack T, B, NK cells and have defects in dendritic and macrophage functions. In addition, a mouse model of endometriosis allowed us to sacrifice animals to harvest whole organs, an option that is not sustainable when using a primate model in the preliminary testing of pharmaceutical agents. Given the high association of thalidomide usage during pregnancy with organ dysgenesis, the histology of the kidney, liver, ovary, and uterus of mice treated with ABT-898 before pregnancy or *in utero* was evaluated. Chronic treatment with ABT-898 did not affect organ morphology or histology in both F0 and F1 generations. Although further studies are warranted to determine the direct effects of ABT-898 on organ function, these findings are the first to suggest that this drug may not affect organ histology.

Angiogenesis is essential to the maintenance of the endometrium, normal ovarian function, and pregnancy [20]. Therefore, we assessed the affects of chronic ABT-898 exposure on physiological angiogenesis. In this study, mice treated with ABT-898 delivered healthy offspring; therefore, it is possible that ABT-898 may not impair physiological angiogenesis in pregnancy. Abnormal plasma levels of angiogenic cytokines could reflect an effect of ABT-898 on angiogenesis. For instance, the detection of reduced VEGF plasma levels in treated mice may indicate that ABT-898 affects physiological angiogenesis regulated by VEGF. However, analysis of IL-15, IL-18, bFGF, LIF, M-CSF, MIG, MIP-2, PDGF-BB, and VEGF plasma levels throughout treatment revealed no significant differences between groups. However, one of the limitations of cytokine analysis is that alymphoid mice lack immune cells (T, B and NK) that are major producers of several cytokines measured in the study. In addition, examination of cellular and vascular structures through series of histological approaches using Haematoxylin and Eosin staining, Periodic Acid Schiff’s reagent staining and immunohistochemistry using vimentin and cytokeratin revealed a conservation of cellular and vascular integrity in ABT-898 treated uteri and ovaries. Electron microscopy of uteri from mice treated chronically with ABT-898 revealed normal capillary structure (unpublished data). ABT-898 did not appear to negatively affect uterine or ovarian function since treated mice were able to achieve and maintain pregnancies. Our lab has also demonstrated that ABT-898 does not affect primordial, primary, secondary, or antral follicle numbers (unpublished data) providing evidence that ABT-898 does not affect ovarian function. These findings indicate that ABT-898 may not impair the maintenance of physiological vessels essential for female reproductive organ function in mice.

For the first time, we have demonstrated of the compatibility of ABT-898 treatment with fertility, trans-generational pregnancy, and offspring development in a murine model of endometriosis. ABT-898 exposure does not impair reproductive status, litter size, or offspring growth across three generations of mice. Furthermore, the anti-angiogenic effects of ABT-898 resulted in an inhibition of pathological angiogenesis within endometriotic lesions while physiological angiogenesis of the uterus and ovary was maintained. Organ dysgenesis was absent in ABT-898 treated mice revealing no obvious histological anomalies in the kidney, liver, ovary, and uterus. As such, our results highlight the potential use of ABT-898 to inhibit endometriotic lesion vascularization without impairing fertility. Future investigations will evaluate the direct effects of ABT-898 on long-term organ function.
